# Exploring the Mediating Role of Emotion Regulation and Distress Intolerance in the Relationship Between Social Anxiety and Problematic Smartphone Use: A Cross‐Sectional Study

**DOI:** 10.1002/hsr2.72356

**Published:** 2026-04-16

**Authors:** Rachel A. Bond, Jon D. Elhai, Christian Montag

**Affiliations:** ^1^ Department of Neurosciences and Psychiatry University of Toledo Toledo, Ohio USA; ^2^ Department of Psychology University of Toledo Toledo, Ohio USA; ^3^ Centre for Cognitive and Brain Sciences Institute of Collaborative Innovation, University of Macau Macau SAR China; ^4^ Department of Computer and Information Science Faculty of Science and Technology, University of Macau Macau SAR China; ^5^ Department of Psychology Faculty of Social Sciences, University of Macau Macau SAR China

**Keywords:** distress intolerance, distress tolerance, emotion regulation, problematic smartphone use, smartphone addiction, social anxiety

## Abstract

**Background and Aims:**

An increasing body of research has demonstrated significant relations between social anxiety and problematic smartphone use (PSU). While some cognitive and affective mechanisms, such as poor emotion regulation, have been explored as mediating variables explaining these relations, there is less known about the role of distress intolerance. Our study explored the mediating role of emotion regulation strategies (expressive suppression and cognitive appraisal) and distress intolerance between social anxiety and PSU severity using structural equation modeling (SEM). We built our research model based upon the framework of the Person‐Affect‐Cognition‐Execution (I‐PACE) theoretical model of Internet use disorders.

**Methods:**

Our sample included 308 undergraduate students aged 18–25 who completed a series of self‐report measures.

**Results:**

SEM results revealed that only expressive suppression significantly mediated the relationship between social anxiety and PSU severity.

**Conclusion:**

Results support prior research that poor emotion regulation skills serve as an underlying mechanism between psychopathology and PSU. Individuals with social anxiety are more likely to engage in expressive suppression, and therefore PSU. Clinical implications include the need to explore the emotional regulation mechanism underlying the relationship between social anxiety and vulnerability to engage in maladaptive behaviors such as PSU.

## 1 Introduction

Excessive smartphone use, also known as problematic smartphone use (PSU), has been associated with several negative health and physical consequences ranging from sleep difficulties [1, 2, 3] to driving and pedestrian accidents [4, 5]. Concerningly, PSU has a well‐established association with increased mental health symptoms, particularly depression and anxiety symptoms broadly [3, 6, 7], and social anxiety more specifically [6, 8]. However, comparatively little research has focused on emotion regulation processes as mechanisms that may explain relations between mental health problems and PSU.

PSU is typically defined as overuse of a smartphone with associated functional impairment‐related consequences [6]. PSU is not considered an official diagnosis in either the Diagnostic and Statistical Manual of Mental Disorders (DSM‐5‐TR [9]) or International Classification of Diseases (ICD‐11 [10]). However, it is nonetheless considered by experts as either an addictive behavior or mobile form of “Internet Use Disorder” with detrimental consequences [11, 12]. This conceptualization of PSU is also in‐line with results from qualitative research studies where adolescents and university students have described PSU as habitual and an “addiction” [13, 14]. And we acknowledge that it is not the smartphone device itself that can be addictive, but rather specific smartphone applications (e.g., social media and messengers [15, 16]), in a similar vein to how an alcoholic is not addicted to the bottle but rather to its content [11]. Nonetheless, the focus of studying PSU makes sense if researchers are interested in studying the effects of the device with its many distractors.

PSU has been associated across the literature with depression and anxiety symptom severity with moderate effect sizes [3, 7, 8, 17]. In particular, PSU and social anxiety disorder (SAD) severity are associated across studies [8]. However, more recently, research has moved beyond examining traditional mental health symptoms related to PSU severity. Specifically, researchers have recently explored cognitive and affective process variables that may serve not only as risk factors for PSU but also may mediate associations between mental health symptoms and PSU [18]. Two such cognitive and affective processes relevant to our study are emotion regulation, and the related construct of distress intolerance [17].

Emotion regulation is a multidimensional construct involving how we manage negative emotion. One established conceptualization suggests that emotion regulation has two primary facets: cognitive reappraisal and expressive suppression [19]. Cognitive reappraisal involves utilizing adaptive mindset adjustments to situations and has been linked to positive outcomes such as subjective well‐being and social connections [19, 20]. Expressive suppression, characterized by avoidance of emotional responses, is associated with negative affective, cognitive, and social consequences [19, 20]. Meta‐analytic findings have shown a moderate positive correlation between poor emotion regulation and PSU severity, including a small, but significant expressive suppression‐PSU correlation [21]. Furthermore, several studies have demonstrated poor emotion regulation as a mediator in the relationship between psychopathology and PSU severity [22, 23, 24]). For example, Elhai et al. [22] found expressive suppression mediated the relationship between anxiety and PSU severity, and Zsido et al. [23] found that maladaptive emotion regulation strategies mediated the link between social anxiety and PSU severity.

Relevant to emotion regulation, distress tolerance is defined as one's perceived ability to endure negative internal states, including negative emotions [25]. While emotion regulation encompasses a broader range of cognitive and behavioral processes to cope with negative emotions, distress tolerance is a distinct, though related, process with several aspects of experiencing negative emotions including tolerance of emotional distress, perception of acceptability of emotional distress, attentional indifference to emotional distress, and use of emotion regulation to relieve distress [25]. Said differently, distress tolerance influences both one's willingness to experience emotional discomfort and one's ability to cope with it adaptively through emotion regulation. Distress tolerance is considered adaptive and is inversely related to anxiety and anxiety sensitivity [26, 27], as well as depressive symptoms [24, 25]. Prior research has found that increased distress intolerance was associated with less ability to control Internet use [28, 29, 30], and specifically more distress intolerance was related to greater PSU severity [26].

The Interaction of Person‐Affect‐Cognition‐Execution (I‐PACE) theoretical model of Internet use disorders posits that predisposing factors, such as biology, genetics, deep‐seated cognitive biases, and mental health disorders, contribute to the development and persistence of specific Internet use disorders, including PSU [31, 32]. I‐PACE also conceptualizes that responses to predisposing variables, such as cognitive and affective processes, coping styles, and poor executive functioning, also serve as risk factors for Internet overuse [32]. Furthermore, cognitive and affective response variables are considered in I‐PACE as mechanisms that may account for relations between predisposing variables and Internet use disorders.

While various forms of psychopathology and mental health disorders have demonstrated a relationship with PSU in prior literature, our interest in the present paper is the study of social anxiety. People with social anxiety are more likely to engage in processes like expressive suppression (i.e., emotion dysregulation), and might prefer to communicate via online channels to avoid direct person‐to‐person contact [22, 23, 33]. By constantly avoiding direct interactions and instead increasingly relying on online communication, likely via the smartphone, excessive online use patterns might be established. In this realm, research on autistic traits and overuse of the Internet is also of interest [34, 35]. Emotion regulation and distress tolerance would represent cognitive and affective response variables in I‐PACE. Poor emotion regulation and greater distress intolerance would serve as risk factors for PSU, and could explain relations between psychopathology (such as social anxiety) and PSU severity. Further elucidation of these relations could also provide important clinical considerations, such as whether it is important to assess for PSU in individuals seeking psychological treatment for conditions such as social anxiety disorder.

Our aim was therefore to examine two variables related to managing and dealing with emotion ‐ emotion regulation and distress intolerance — in association with PSU. We were also interested in examining the mediating role of emotion regulation and distress intolerance between social anxiety and PSU severity. We posed the following hypotheses:

Social anxiety would be related to greater PSU severity [8].

Emotion regulation would be associated with PSU severity [21]. Specifically, cognitive appraisal would be related to decreased, but expressive suppression would be related to increased PSU.

Increased distress intolerance would be related to greater PSU severity [26, 28, 29, 30].

Emotion regulation would mediate relations between social anxiety and PSU severity [22, 23].

Greater distress intolerance would mediate relations between social anxiety and PSU severity. For this hypothesis, we did not have prior work from which to draw. However, because distress tolerance is similar to emotion regulation, and since emotion regulation mediated relations between social anxiety and PSU severity [22, 23], it is reasonable that this hypothesis would be supported.

### 1.1 Research Model

**Figure 1:**
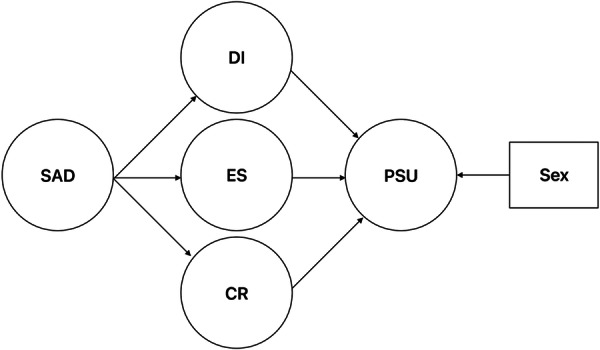
Structural Equation Model with Emotion Regulation Variables and Distress Intolerance Mediating the Relationship between Social Anxiety and PSU, Controlling for Sex. Latent variables are represented by circles; observed variables are represented by rectangles. CR = cognitive reappraisal, DI = distress intolerance, ES = expressive suppression, PSU = problematic smartphone Use, SAD = social anxiety disorder.

Figure 1 displays our research model, with social anxiety specified to predict distress intolerance, emotional expressive suppression, and cognitive reappraisal; these mediators are then specified to predict PSU. We controlled for sex in paths leading to PSU, because sex is often related to PSU severity [36]. Given the relatively homogenous age group (discussed next), we did not control for age.

## 2 Methods

### 2.1 Procedure and Participants

We recruited a total of 437 undergraduate students aged 18–25 who endorsed currently owning a smartphone, and were enrolled in an introductory psychology course at a mid‐sized Midwestern US university. The study was advertised with a brief description via the Psychology Department's Sona Systems research participation web portal, as students are required to participate in research activities. The study was first approved by the university's Institutional Review Board.

Upon providing informed consent online, participants were directed to an online, self‐report Qualtrics survey which took approximately 10–15 min to complete. We removed 61 participants for duplicate entries based on the timestamp of completion and the Qualtrics system ID. An additional 29 participants were removed based on age exclusion criteria, and another 39 participants due to careless responding (with the same answer to 17+ consecutive questions). The final sample included 308 participants.

Participants' average age was 19.46 (SD = 1.48), and a slight majority reported biological sex as female (*n* = 194, 62.78%). A little over two‐thirds reported Caucasian/White racial background (*n* = 201, 65.26%), with some racial and ethnic representation from Latinx (*n* = 50, 16.23%), Asian (*n* = 56, 18.12%), and African American/Black (*n* = 31, 10.03%) participants. Over half of participants reported being freshman (*n* = 188, 60.84%), and about half reported working part‐time (*n* = 156, 50.49%).

### 2.2 Measures

In addition to demographics, participants completed a battery of self‐report questionnaires in English. Coefficient *α* estimates in our sample are provided in Table 1; we also present McDonald's Omega values (for ordinal items).

**Table 1:** Internal consistency and descriptive statistics of primary variables by sex.

Variable	Alpha/Omega	Men *M* (SD)	Women *M* (SD)	Sex *F*(1,307)	*p*	*η* ^2^_*p*_
SAD	0.95/0.96	22.79 (14.99)	29.55 (16.42)	13.02	< 0.001	0.04
ES	0.70/0.76	17.29 (4.40)	15.54 (4.70)	10.44	0.001	0.03
CR	0.82/0.85	28.11 (6.53)	29.66 (6.19)	4.35	0.04	0.01
DI	0.91/0.93	39.20 (11.19)	42.64 (11.38)	6.68	0.01	0.02
PSU	0.86/0.89	26.10 (9.58)	29.57 (9.94)	9.00	0.003	0.03

Abbreviations: DI = distress intolerance, ES = expressive suppression, PSU = problematic smartphone use, SAD = social anxiety disorder.

*Distress Tolerance Scale (DTS)*. The DTS [37] is a 15‐item self‐report measure of emotional distress tolerance, using response options from 1 (strongly agree) to 5 (strongly disagree) with total scores ranging from 15 to 75. We deviated from the original scale's instructions by instead using a 1 (strongly disagree) to 5 (strongly agree) scale, so that total scores generated from summing items, after reverse‐coding one items, indicate greater distress intolerance (DI) to allow for enhanced ease of interpretation as distress intolerance is of more relevance to the current study. The DTS has good validity and reliability [38].

*Emotion Regulation Questionnaire (ERQ)*. The ERQ (Gross & John, 2003) assesses differences in emotion regulation strategies with 10 self‐report items rated on a Likert scale from 1 (strongly disagree) to 7 (strongly agree). We used a past‐month time‐frame. Although item scoring is continuous, its facets are scored and interpreted separately; thus, we summed relevant items to form the cognitive appraisal (1, 3, 5, 7, 8, and 10) and expressive suppression (items 2, 4, 6, and 9) subscales. Higher scores from either subscale indicate greater engagement in that emotion regulation strategy. The ERQ has demonstrated criterion validity and reliability [39].

*Smartphone Addiction Scale‐Short Version (SAS‐SV)*. The SAS‐SV [40] is a 10‐item measure that assesses PSU, with a response scale from 1 (strongly disagree) to 6 (strongly agree) with total scores ranging from 10 to 60. Higher scores indicate greater PSU. It has good psychometric properties [40, 41].

*Social Interaction Anxiety Scale (SIAS)*. The SIAS [42] measures social interaction anxiety using 20 items answered on a Likert scale from 1 (not at all characteristic or true of me) to 5 (extremely characteristic or true of me). We used a past‐month time‐frame. The SIAS is typically scored by only summing the 17 non‐reverse‐coded items for a total score ranging from zero to 68, with good psychometrics [43]. Higher scores indicate greater social interaction anxiety.

### 2.3 Analyses

All data screening procedures, preliminary analyses, zero‐order correlations, and one‐way ANOVAs were conducted with R software [44]. The following R packages were used during data pre‐processing and preliminary analyses: *careless* to detect careless responding; *mice* for missing data; *fmsb* for internal consistency; *pastecs* for descriptives; *sjstats* and *car* for ANOVA; and *corrplot* for correlations. Missing data at the item‐level were imputed using the expectation maximization algorithm, one scale at a time before computing scale scores. Data did not indicate substantial non‐normality, with the largest skewness and kurtosis values being 1.13 and 1.33, respectively.

Data were first analyzed using bivariate correlations for scale scores and age, as well as ANOVAs to examine scale score differences by sex. Please note that age correlations need to be considered restricted given the small variance in age among participants. Confirmatory factor analysis (CFA) and structural equation modeling (SEM) were conducted using Mplus v.8.10 [45]. For emotion regulation (which uses a 7‐point continuous response scale), we used maximum likelihood estimation with robust standard errors (MLR), a Pearson covariance matrix and linear regression‐based factor loadings to estimate a two‐factor CFA of cognitive reappraisal and expressive suppression. The remaining measures (SAS‐SV, DTS, SIAS) use response scales with six or fewer options and with descriptive anchors for each numerical response option. So, for those three measures, we treated items as ordinal, utilizing weighted least squares estimation with a mean‐and‐variance‐adjusted chi‐square (WLSMV), estimating a polychoric covariance matrix, with factor loadings computed with probit regression [46], to test single‐factor CFAs of PSU, distress intolerance, and SAD. For PSU, because of extremely similar item content between its first two items, and between items 4 and 5, we specified a residual error covariance between each of those two item pairs [47, 48].

We subsequently tested the SEM pictured in Figure 1, using WLSMV estimation. Mediation effects were tested by computing the cross‐products of direct path coefficients with non‐parametric bootstrapping procedure using 1,000 iterations for more accurate estimates [49]. Standard errors (SE) were estimated with the Delta method.

## 3 Results

### 3.1 Preliminary Results

**Figure 2:**
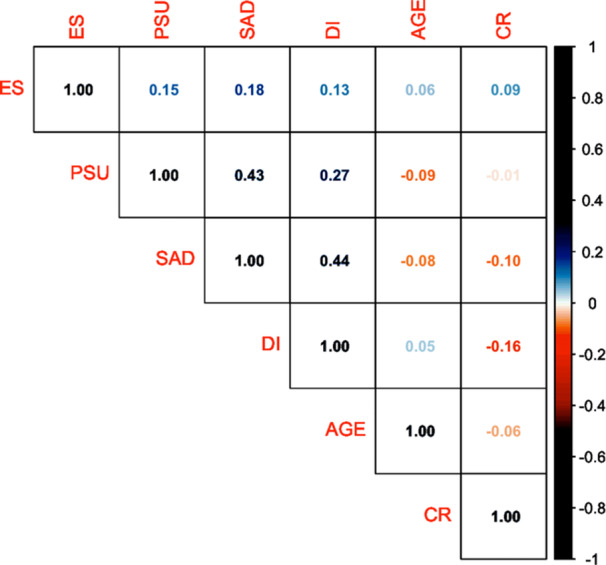
Correlation heat map of primary variables. CR = cognitive reappraisal, DI = distress intolerance, ES = expressive suppression, PSU = problematic smartphone use, SAD = social anxiety disorder. Darker values indicate stronger correlations (but pay attention to whether the sign is positive or negative). In the online version of this article, red‐colored numbers indicate negative associations, while blue‐colored numbers indicate positive associations. Correlations greater than 0.10 in absolute size were *p* < 0.05; correlations greater than 0.15 in absolute size were *p* < 0.01.

Descriptive statistics, and one‐way ANOVA results by sex, are reported in Table 1. All study variables were significantly related to sex; higher scores were observed for women in all but expressive suppression. However, all effect sizes were small. Zero‐order Pearson correlations between study variables are visualized with a heat map in Figure 2. PSU severity was associated with greater social anxiety (H1) and distress intolerance (H3) with at least moderate effect sizes. PSU severity was related to greater expressive suppression (H2) with a small effect, but was not significantly associated with cognitive reappraisal (H2). Age was not significantly related to any scale scores.

### 3.2 CFA & SEM Results

The single‐factor PSU measurement model yielded adequate fit, WLSMV *χ* ^2^(33) = 209.93, *p* < 0.001, CFI = 0.92, TLI = 0.90, RMSEA = 0.13 (90% CI: 0.12–0.15), SRMR = 0.05. The single‐factor social anxiety model yielded excellent fit, WLSMV *χ* ^2^(119) = 379.43, *p* < 0.001, CFI = 0.98, TLI = 0.97, RMSEA = 0.09 (90% CI: 0.08–0.09), SRMR = 0.04. The single‐factor distress intolerance model demonstrated adequate fit, WLSMV *χ* ^2^(90) = 596.54, *p* < 0.001, CFI = 0.91, TLI = 0.89, RMSEA = 0.14 (90% CI: 0.13–0.15), SRMR = 0.06. And the two‐factor emotion regulation model yielded adequate fit, MLR *χ* ^2^(34) = 69.22, *p* < 0.001, CFI = 0.95, TLI = 0.93, RMSEA = 0.06 (90% CI: 0.04–0.08), SRMR = 0.06. RMSEA did not demonstrate evidence of acceptable fit for PSU, social anxiety, or distress intolerance, however this is expected with ordinal items [50].

**Figure 3:**
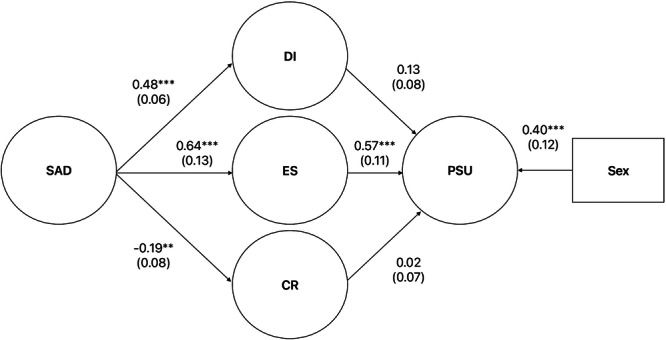
Standardized estimates of SEM*.* ***p < 0.001, **p = 0.01, *p = 0.05. Numbers reported in parentheses are standard errors. Latent variables are represented by circles; observed variables are represented by rectangles. CR = cognitive reappraisal, DI = distress intolerance, ES = expressive suppression, PSU = problematic smartphone use, SAD = social anxiety disorder. Sex was coded male = 1, female = 2. Factor loadings are not displayed in this figure, to reduce clutter, but are displayed in Table 2. Residual error covariances are available upon request from the corresponding author.

The SEM from Figure 1 was tested next. Standardized pathways are illustrated in Figure 3 below. Results demonstrated adequate, though not excellent, fit, *χ* ^2^(1317) = 2356.56, *p* < 0.001, CFI = 0.92, TLI = 0.92, SRMR = 0.09, RMSEA = 0.05 (90% CI: 0.05–0.05). Adjusting for sex, PSU severity was significantly related to greater expressive suppression (H2), but not to distress intolerance (testing H3) or cognitive reappraisal (H2). PSU was related to female sex. Social anxiety was significantly related to greater distress intolerance, poorer cognitive reappraisal, and greater emotional expressive suppression. Factor loadings for all CFAs are summarized in Table 2.

**Table 2:** Factor loadings of latent variables.

Factor	Item	Loading	SE	*p*
DI	DTS1	0.65	0.04	< 0.001
	DTS2	0.78	0.04	< 0.001
	DTS3	0.80	0.30	< 0.001
	DTS4	0.83	0.03	< 0.001
	DTS5	0.57	0.05	< 0.001
	DTS6	0.39	0.08	< 0.001
	DTS7	0.62	0.05	< 0.001
	DTS8	0.45	0.06	< 0.001
	DTS9	0.76	0.03	< 0.001
	DTS10	0.79	0.03	< 0.001
	DTS11	0.72	0.04	< 0.001
	DTS12	0.80	0.03	< 0.001
	DTS13	0.56	0.05	< 0.001
	DTS14	0.43	0.07	< 0.001
	DTS15	0.80	0.03	< 0.001
CR	ERQ1	0.46	0.10	< 0.001
	ERQ3	0.64	0.08	< 0.001
	ERQ5	0.73	0.01	< 0.001
	ERQ7	0.75	0.06	< 0.001
	ERQ8	0.82	0.06	<;0.001
	ERQ10	0.74	0.07	< 0.001
ES	EQR2	0.24	0.09	0.010
	ERQ4	0.31	0.10	0.001
	ERQ6	0.28	0.10	0.006
	ERQ9	0.28	0.01	0.002
PSU	SAS‐SV1	0.62	0.06	< 0.001
	SAS‐SV2	0.62	0.05	< 0.001
	SAS‐SV3	0.52	0.08	< 0.001
	SAS‐SV4	0.73	0.05	< 0.001
	SAS‐SV5	0.82	0.05	< 0.001
	SAS‐SV6	0.76	0.04	< 0.001
	SAS‐SV7	0.75	0.04	< 0.001
	SAS‐SV8	0.64	0.05	< 0.001
	SAS‐SV9	0.59	0.06	< 0.001
	SAS‐SV10	0.69	0.05	< 0.001
SAD	SIAS1	0.73	0.03	< 0.001
	SIAS2	0.78	0.03	< 0.001
	SIAS3	0.69	0.04	< 0.001
	SIAS4	0.73	0.04	< 0.001
	SIAS6	0.74	0.04	< 0.001
	SIAS7	0.81	0.03	< 0.001
	SIAS8	0.65	0.04	< 0.001
	SIAS10	0.79	0.03	< 0.001
	SIAS12	0.77	0.03	< 0.001
	SIAS13	0.56	0.05	< 0.001
	SIAS14	0.61	0.05	< 0.001
	SIAS15	0.86	0.02	< 0.001
	SIAS16	0.86	0.02	< 0.001
	SIAS17	0.86	0.02	< 0.001
	SIAS18	0.80	0.03	< 0.001
	SIAS19	0.88	0.02	< 0.001
	SIAS20	0.77	0.03	< 0.001

Abbreviations: CR = cognitive reappraisal, DI = distress intolerance, DTS = Distress Tolerance Scale, ERQ = Emotion Regulation Questionnaire, ES = expressive suppression, PSU = problematic smartphone use, SAD = social anxiety disorder, SAS‐SV = Smartphone Addiction Scale–Short Version, SIAS = Social Interaction Anxiety Scale.

### 3.3 Mediation Results

Three indirect effect tests were analyzed with mediation (adjusting for sex) including: (a) distress intolerance between social anxiety and PSU (H5); (b) cognitive reappraisal between social anxiety and PSU (H4); and (c) expressive suppression between social anxiety and PSU (H4). Only expressive suppression significantly mediated the relationship between social anxiety and PSU severity. See Table 3 for a summary of standardized estimates for the indirect effects.

**Table 3:** Standardized estimates of indirect/mediation effects.

Mediator variable(s)	Predictor variable	Outcome variable	*β*	SE	*p*
DI	SAD	PSU	0.06	0.04	0.13
CR	SAD	PSU	−0.00	0.01	0.79
ES	SAD	PSU	0.37	0.07	< 0.001

Abbreviations: CR = cognitive reappraisal, DI = distress intolerance, ES = expressive suppression, PSU = problematic smartphone use, SAD = social anxiety disorder.

### 3.4 Model Alteration

Finally, we tested an alteration to the model displayed in Figure 1. Specifically, we modified that model by adding a path directly from social anxiety to PSU. The model fit significantly better than the model from Figure 1, *χ* ^2^_diff_(1) = 37.53, *p* < 0.001. However, the increase in CFI units was less than the typical benchmark of 0.01—specifically, only 0.005 points (from CFI of 0.923 for Figure 1's model to CFI of 0.927 for the model alteration), demonstrating that the model alteration's fit increase was not substantial in magnitude or meaningful. Also, the path coefficients remained virtually the same as in Figure 3.

## 4 Discussion

We tested mediating effects for distress intolerance and emotion regulation variables in the relationship between social anxiety and PSU severity, while controlling for sex. We expected to find significant mediation results based upon prior literature supporting mediating roles of distress intolerance and/or emotion regulation between anxiety and PSU severity [22, 23, 28, 29, 30, 51]. Our mediation tests were also consistent with principles of the I‐PACE model [32].

Regarding our hypotheses, H1 was supported, with social anxiety related to greater PSU severity based on Pearson correlations (see Figure 2). This result was expected and supports prior research finding similar results [8, p. 2) and based on I‐PACE with social anxiety being a type of psychopathology associated with an Internet‐use disorder such as PSU [32].

For H2, we predicted that emotion regulation would be associated with PSU severity, with more adaptive strategies (i.e., cognitive reappraisal) being related to decreased PSU and maladaptive strategies (i.e., expressive suppression) related to increased PSU. Our results show some support for H2, with expressive suppression being related to increased PSU severity via Pearson correlations, though cognitive reappraisal was not (see Figure 2). Our SEM revealed a similar pattern, where expressive suppression, but not cognitive reappraisal, was related to PSU severity after controlling for covariates. Overall, these results align with prior findings by Shahidin et al., [21] and fit within I‐PACE where poor emotion regulation could be considered a maladaptive cognitive or affective process that could increase risk for the development PSU or other Internet use disorders [32]. We would describe emotional regulation as a top‐down process, hence where cortical activity is needed to downregulate subcortical energy in light of emotional turmoil (for instance see Panksepp's Affective Neuroscience Theory on primary emotional systems [52, 53]). Against this background, poor emotional regulation processes might therefore be better characterized as maladaptive cognitive processes, although overactivity in emotional parts of the brain in social situations show that affective processes are also part of the equation.

We predicted in H3 that greater distress intolerance would relate to greater PSU severity, supported through Pearson correlations with around a medium effect and aligns with prior research [22, 28, 29, 30, 51]. However, in the SEM after controlling for covariates, distress intolerance was not significantly related to PSU severity. Distress intolerance is quite related to poorer emotion regulation abilities. Emotion dysregulation has been demonstrated as a facet of distress intolerance in factor analysis [54]. In our sample, Pearson correlations aligns with this conceptualization, as greater distress intolerance had small, but significant relations with greater expressive suppression (i.e., emotion dysregulation) and poorer cognitive appraisal (i.e., emotion regulation). This interrelationship between distress tolerance and emotion regulation strategies could explain why distress tolerance was only bivariately, but not multivariately, related to PSU severity as demonstrated in prior research [25, 28, 30, 54].

For our fourth hypothesis (H4), we predicted that emotion regulation would mediate relations between social and anxiety and PSU severity. Our results revealed that expressive suppression (i.e., emotion dysregulation) was a significant mediator, but not cognitive reappraisal (i.e., adaptive emotion regulation), supporting prior research [22, 23]. Expressive suppression being such a mediator fits well with I‐PACE where poor emotion regulation could serve as a mechanism explaining why some people with psychopathology, such as social anxiety, may engage in PSU [32].

Our final hypothesis (H5) was not supported, as distress intolerance did not mediate relations between social anxiety and PSU severity. We could not locate prior work testing this research question, but the null finding was contrary to our expectations given the work discussed above finding that emotion regulation mediates relations between social anxiety and PSU [22, 23]; after all, distress intolerance and poor emotion regulation are quite related conceptually and empirically [54]. The null mediation finding for distress intolerance may be explained by the inclusion of the two emotion regulation strategies as covariates of PSU, given that distress tolerance and emotion regulation covary.

Overall, results support prior literature identifying a relationship between SAD and PSU [8, p. 2] and the mediating role of emotion dysregulation (i.e., expressive suppression) in this relationship [22, 23]. These results elicit important clinical implications and considerations regarding treatment of SAD, especially given prior research identifying a relationship between social anxiety symptom severity and expressive suppression [55], potential preference of online communication via smartphone to avoid direct in‐person contact [33], and increased risk of those diagnosed with SAD developing PSU based on principles of the I‐PACE model [32, 56]. Said differently, given the increased likelihood for PSU to develop in those with SAD, it is important to assess for PSU in individuals seeking treatment for social anxiety in clinical settings. PSU can be conceptualized as a maladaptive, emotion regulation‐based coping strategy, for which well‐established, empirically‐validated psychotherapeutic treatments such as cognitive behavioral therapy (CBT [57]) could be employed to target. Specifically, for those with social anxiety disorder, utilizing exposure‐based interventions targeting PSU might be particularly effective [58].

The present study had some limitations that future research should address, including the use of a non‐clinical sample of convenience of undergraduate students from the United States collected using cross‐sectional methods. Cross‐sectional methods do not allow for testing the true directionality of research variables. And it is plausible that poor emotion regulation may influence distress intolerance, rather than being covaried as modeled in this paper. It is also important to consider that results from our sample are likely not universally generalizable to non‐Western and clinical populations [59, 60], though future research including more diverse sampling and cross‐cultural research could be beneficial in further conceptualizing the relationship between psychopathology and PSU. Also, we relied on self‐report measures which are fallible ways of measuring psychopathology and PSU. In future research, a clinical sample might better capture constructs such as social anxiety, using structured interviewing and objective methods, and experimental or longitudinal approaches could allow for assessment of directionality of variables. Despite limitations, the study had notable strengths including targeting an age group specifically associated with smartphone the use, and using a sample size sufficient for use of robust statistical analyses [17, 61].

## Author Contributions

**Rachel A. Bond:** conceptualization, methodology, data curation, project administration, formal analysis, writing – original draft. **Jon D. Elhai:** conceptualization, methodology, supervision, formal analysis, writing – review and editing. **Christian Montag:** writing – review and editing.

## Funding

The authors have nothing to report.

## Ethics Statement

IRB approval was received from the University of Toledo's IRB before the project was conducted. The procedures used in this study adhere to the tenets of the Declaration of Helsinki. Informed consent was obtained from all individual participants included in the study.

## Consent

All participants provided consent via an informed consent statement before participating.

## Conflicts of Interest

The authors report no conflicts of interest with this paper's study. However, outside the scope of the present paper, the authors report the following:

Dr. Elhai notes that he receives royalties for several books published on posttraumatic stress disorder (PTSD); occasionally serves as a paid, expert witness on PTSD legal cases; and has recently received research grant funding from the U.S. National Institutes of Health.

For reasons of transparency Dr. Montag mentions that he has received (to Ulm University and earlier University of Bonn) grants from agencies such as the German Research Foundation (DFG). Dr. Montag has performed grant reviews for several agencies; has edited journal sections and articles; has given academic lectures in clinical or scientific venues or companies; and has generated books or book chapters for publishers of mental health texts. For some of these activities he received royalties, but never from gaming or social media companies. Dr. Montag mentions that he was part of a discussion circle (Digitalität und Verantwortung: https://about.fb.com/de/news/h/gespraechskreis-digitalitaet-und-verantwortung/) debating ethical questions linked to social media, digitalization and society/democracy at Facebook. In this context, he received no salary for his activities.

## Transparency Statement

The corresponding author Jon D. Elhai affirms that this manuscript is an honest, accurate, and transparent account of the study being reported; that no important aspects of the study have been omitted; and that any discrepancies from the study as planned (and, if relevant, registered) have been explained.

## Data Availability

The data that support the findings of this study are available from the corresponding author upon reasonable request.
